# The Rectum in Its Relation to Uterine Disease

**Published:** 1875-03

**Authors:** Arthur W. Edis


### THE RECTUM IN ITS RELATION TO UTERINE DISEASE.

BY ARTHUR W. EDIS, M. D.

The author was desirous of drawing attention to the important help afforded us by the rectum, both as to the diagnosis and treatment of uterine disorders. Although known to the few, exploration by the rectum seemed scarcely as generally known as its importance would suggest; and, for the relief of pelvic symptoms, there were numerous cases where sedatives applied *per rectum* were of far greater service than when taken in the ordinary way. As regards the diagnosis of uterine disorders, there were several methods of rectal exploration : viz., the ordinary digital one—useful in cases of retroflexion, fibroid, and ovarian tumors, &c.; eversion of the rectum through its sphincter, like the finger of a glove, by pressure from the vagina; manual exploration, as suggested by Dr. Gustave Simon. In reference to the treatment of uterine disorders by the aid of the rectum, the cases were very numerous. Reposition of the retroflexed gravid uterus could readily be accomplished by pressure *per rectum.* The relief of imagined uterine symptoms might often be effected by a proper attention to the condition of the bowels. The question of absorption by the rectum was too well recognized to need discussion. Pessaries and suppositories made of gelatin and glycerin were far more useful than those made with cocoa-butter, absorption being more uniform and certain. In menorrhagia, enemata of ice-cold water, acting by reflex action, proved of great service. In dysmenorrhœa, enemata or suppositories of morphia, belladonna, and other agents, were very useful, and too little resorted to. In the morning sickness of pregnancy, the most intractable vomiting might often be relieved by opiate enemata or suppositories, and life sustained for many weeks by nutrient enemata. In cancer of the uterus, suppositories were far more useful than pessaries in allaying pain. In chronic constipation, small suppositories of soap or extract of belladonna were of much service. As to diseases of the rectum itself being mistaken for uterine disorder, the mistake was not unfrequent; fissure causing vaginimus, ulcer causing dyspareunia, menorrhagia, &c.—*New Orleans Medical and Surgical Journal.*

